# Molecular characterization of woodchuck IFI16 and AIM2 and their expression in woodchucks infected with woodchuck hepatitis virus (WHV)

**DOI:** 10.1038/srep28776

**Published:** 2016-06-29

**Authors:** Qi Yan, Mengmeng Li, Qin Liu, Fanghui Li, Bin Zhu, Junzhong Wang, Yinping Lu, Jia Liu, Jun Wu, Xin Zheng, Mengji Lu, Baoju Wang, Dongliang Yang

**Affiliations:** 1 Department of Infectious Diseases, Union Hospital, Tongji Medical College, Huazhong University of Science and Technology, Wuhan, China; 2 Institute of Virology, University of Duisburg-Essen, Essen, Germany

## Abstract

IFI16 and AIM2 are important DNA sensors in antiviral immunity. To characterize these two molecules in a woodchuck model, which is widely used to study hepatitis B virus (HBV) infection, we cloned and analyzed the complete coding sequences (CDSs) of woodchuck IFI16 and AIM2, and found that AIM2 was highly conserved in mammals, whereas the degree of sequence identity between woodchuck IFI16 and its mammalian orthologues was low. IFI16 and IFN-β were upregulated following VACV ds 70 mer transfection, while AIM2 and IL-1β were upregulated following poly (dA:dT) transfection, both *in vitro* and *in vivo*; IFI16-targeted siRNA decreased the transcription of IFI16 and IFN-β stimulated by VACV ds 70 mer, and AIM2 siRNA interference downregulated AIM2 and IL-1β transcripts stimulated by poly (dA:dT), *in vitro*, suggesting that woodchuck IFI16 and AIM2 may play pivotal roles in the DNA-mediated induction of IFN-β and IL-1β, respectively. IFI16 and AIM2 transcripts were upregulated in the liver and spleen following acute WHV infection, while IFI16 was downregulated in the liver following chronic infection, implying that IFI16 and AIM2 may be involved in WHV infection. These data provide the basis for the study of IFI16- and AIM2-mediated innate immunity using the woodchuck model.

Hepatitis B virus (HBV), a member of the *hepadnaviridae* family, is a small DNA virus that causes acute and chronic hepatitis B^1^. Approximately 240 million people are chronically infected with HBV worldwide and have an increased risk for hepatocellular carcinoma (HCC) (http://www.who.int/mediacentre/factsheets/fs204/en/). More than 1 million people die annually from chronic HBV-related diseases^2^,^3^. Innate immunity is important in controlling the spread of the pathogen and developing an adaptive immune response. It is widely accepted that HBV-specific T cell responses are responsible both for viral clearance and for disease pathogenesis^4^, although the sensing of HBV by the innate immune system is not well understood^5^. HBV covalently closed circular DNA (cccDNA), a transcriptional template for the synthesis of viral RNA during HBV replication, is localized to the nucleus of a hepatocyte; thus, intracellular DNA sensors may be candidates for sensing HBV^6^.

Interferon γ-inducible protein 16 (IFI16) and absent in melanoma 2 (AIM2) are two members of the AIM2-like receptor (ALR) family^7^,^8^,^9^,^10^,^11^,^12^. ALRs share a partially conserved hematopoietic interferon-inducible nuclear antigens with a 200-amino-acid repeat (HIN-200) domain in the C-terminus that detects double-stranded DNA (dsDNA)^13^,^14^. Most ALRs also contain a homotypic protein-protein interaction Pyrin domain in the N-terminus that binds the downstream adaptor. IFI16 detects intracellular DNA and induces interferon-β (IFN-β) by regulating the activation of interferon regulatory factor 3 (IRF3) and nuclear factor-kappa B (NF-κB)^15^,^16^,^17^. IFI16 was found to be critical for restricting the replication of many viruses, including human immunodeficiency virus (HIV), cytomegalovirus (CMV) and Kaposi sarcoma-associated herpes virus (KSHV)^18^,^19^. AIM2 detects cytoplasmic dsDNA, associates with the apoptosis-associated speck-like protein containing a CARD (ASC) and promotes the caspase-1-dependent production of interleukin-1β (IL-1β)^20^,^21^,^22^,^23^. AIM2 plays an important role in sensing both bacterial and DNA viral pathogens^24^,^25^,^26^.

Although the chimpanzee is the only animal that can be infected by HBV, the use of this animal is expensive and ethically limited^27^. Woodchuck hepatitis virus (WHV) is a member of the *hepadnaviridae* family and can infect the American woodchuck (*M. monax*) and the Chinese woodchuck (*M. himalayana*). Woodchuck WHV infection strongly resembles human HBV infection in its major virological, pathological, and immunological features^1^,^28^,^29^,^30^. Hence, the woodchuck model is valuable for studying the pathogenesis of HBV infection, evaluating antiviral drugs and developing rational immunotherapeutic strategies^27^,^29^,^31^,^32^. However, the intracellular innate DNA sensors IFI16 and AIM2 require further study in the woodchuck model.

In this study, we characterized the complete coding sequences (CDSs) of woodchuck IFI16 (wIFI16) and AIM2 (wAIM2), examined their roles in IFN-β and IL-1β induction in woodchuck fibroblastoma cells, respectively, and evaluated the effect of their respective ligands, VACV ds 70 mer and poly (dA:dT), *in vivo*. Additionally, we measured the expression of wIFI16 and wAIM2 transcripts in different organs from naïve woodchucks and in the liver and spleen of woodchucks with different WHV-infected status.

## Results

### Sequence analysis of wIFI16 and wAIM2

**Figure 1:**
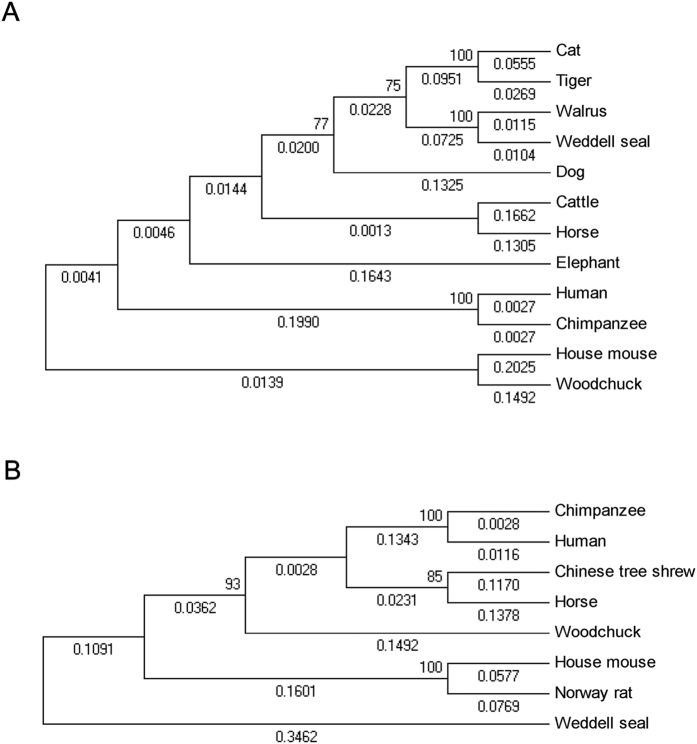
The phylogenetic trees of wIFI16 and wAIM2. Neighbor-joining phylogenetic trees were constructed with the amino acid sequences of wIFI16 (**A**) and wAIM2 (**B**) and their orthologues from other species. The accession numbers of the sequences used for the phylogenetic analysis are listed in Tables 1 and 2.

**Figure 2:**
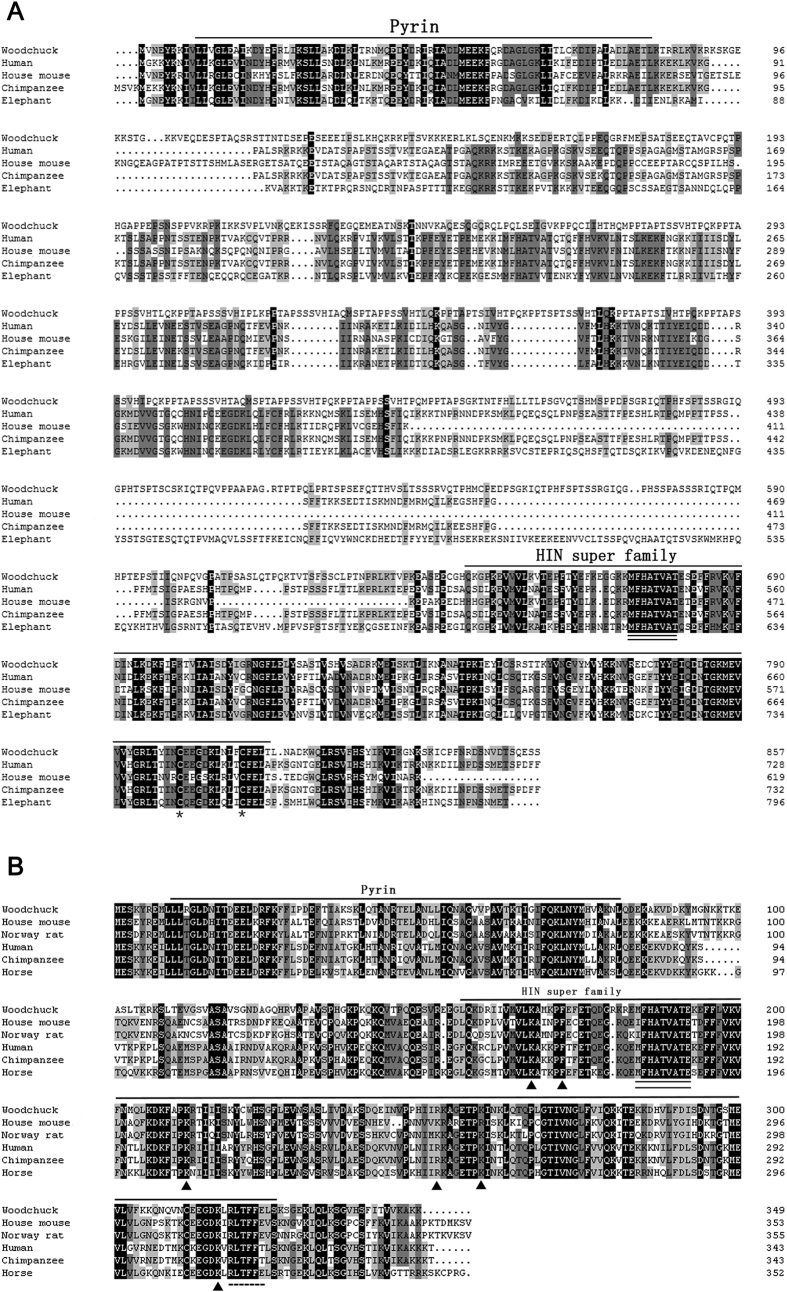
Alignment of the predicted wIFI16 and wAIM2 residues with their orthologues from other species. The accession numbers used for the alignment are listed in Tables 1 and 2. The conserved residues of wIFI16 (**A**) and wAIM2 (**B**) are shaded in black, dark grey and light grey. Dashes indicate gaps in the alignment. Cysteine residues in the HIN domain are denoted with asterisks (*). The conserved MFHATVAT/M(I)HATVAT motif and Rb-binding motifs are indicated with a double-underline (=) and a dashed line (--), respectively. The residues critical for dsDNA binding in the HIN domain are labeled with triangles (▲). The Pyrin and HIN domains are indicated with a line.

The CDS of wIFI16 was submitted to GenBank (accession number KP334127) and consists of 2574 bp with a deduced protein length of 857 amino acids (aas). The sequence identities between wIFI16 and its orthologues in other mammalian species ranged from 52.53 to 76.26% and from 29.27 to 65.33% at the nucleotide (nt) and aa levels, respectively (Table 1). A phylogenetic tree was constructed using the sequences of IFI16 from woodchuck and other mammalian species, indicating that wIFI16 was closely related to mouse IFI16 (Fig. 1A). The Conserved Domain Search Service (CD Search) indicated that wIFI16 contains an N-terminal Pyrin domain (10-83aa) and a C-terminal DNA-binding HIN domain (647-815aa), and the MFHATVAT motif was conserved in the HIN domain (Fig. 2A)^33^. The alignment analysis found 2 cysteine residues (Cys801 and Cys811) that were conserved in the HIN domain of woodchucks and other mammalian species (Fig. 2A).

The CDS of wAIM2 was submitted to GenBank (accession number KP272148) and is 1050 bp in length. The deduced protein has 349 aa residues. The sequence of wAIM2 exhibited high sequence identity with its orthologues from other mammalian species, ranging from 63.58 to 78.14% at the nt level and from 52.80 to 70.09% at the aa level (Table 2). The phylogenetic tree showed that wAIM2 is closest to its human and chimpanzee orthologues (Fig. 1B). The CD Search indicated that there were two conserved domains, the N-terminal Pyrin domain (10-82aa) and the C-terminal DNA-binding HIN domain (157-326aa) in wAIM2, and the M(I)FHATVAT and Rb-binding motifs were conserved (Fig. 2B). A previous study indicated that mutating Lys160, Lys204, Lys251, Lys309, Phe165 or Arg244 in the HIN domain of human AIM2 impairs its binding activity with dsDNA^34^. Accordingly, all of the corresponding sites in the HIN domain of wAIM2 were conserved (Fig. 2B).

### Silencing wIFI16 or wAIM2 impaired IFN-β or IL-1β transcript expression, respectively, in woodchuck fibroblastoma cells

**Figure 3:**
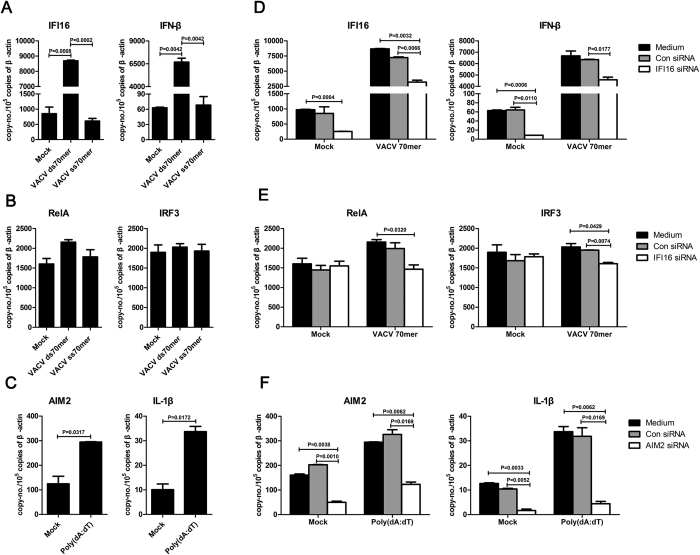
The effects of siIFI16 on IFN-β, and siAIM2 on IL-1β, transcription induction, with or without VACV ds 70 mer or poly (dA:dT) stimulation, respectively. WH12/6 cells were transfected with VACV ds 70 mer (2 μg/ml) or poly (dA:dT) (2 μg/ml) for 24 hours. The wIFI16, wIFN-β (**A**), wRelA, wIRF3 (**B**), wAIM2, and wIL-1β (**C**) were quantified using RT-qPCR. The VACV ss 70 mer (2 μg/ml) was transfected as a control. WH12/6 cells were transfected with siIFI16 or the control siRNAs for 24 hours and then transfected with or without the VACV ds 70 mer (2 μg/ml). Twenty-four hours later, wIFI16, wIFN-β (**D**), wRelA and wIRF3 (**E**) transcripts were quantified using RT-qPCR. WH12/6 cells were transfected with siAIM2 or the control siRNAs for 24 hours and were then transfected with or without poly (dA:dT) (2 μg/ml). Twenty-four hours later, wAIM2 and wIL-1β (**F**) transcripts were quantified using RT-qPCR.

The intracellular VACV ds 70 mer and poly (dA:dT) are known ligands of IFI16 and AIM2, respectively, and can induce IFN-β and IL-1β production, respectively, in human and mice^17^,^21^. To determine whether these two dsDNAs can induce IFN-β and IL-1β production in woodchuck cells, respectively, we examined their mRNA levels in a woodchuck fibroblastoma cell line (WH12/6) with or without VACV ds 70 mer or poly (dA:dT) stimulation. The transcripts of wIFI16 and wIFN-β were dramatically increased, whereas wRelA (a subunit of NF-κB) and wIRF3 were only slightly increased, in VACV ds 70 mer-transfected cells compared with those of the VACV ss 70 mer-transfected or mock-infected cells (Fig. 3A,B). Similarly, wAIM2 and wIL-1β transcripts were upregulated after poly (dA:dT) transfection in WH12/6 cells (Fig. 3C). These data indicate that, at the mRNA level, human and mouse IFI16 and AIM2 ligands can stimulate wIFI16 and wAIM2, respectively, and lead to wIFN-β and wIL-1β production, respectively.

To assess whether intracellular VACV ds 70 mer-induced wIFN-β production was mediated by wIFI16, we measured wIFI16, wRelA, and wIRF3 expression in WH12/6 cells after wIFI16-targeted siRNA interference. Similarly, to determine whether poly (dA:dT)-induced wIL-1β production was mediated by wAIM2, we measured wAIM2 and wIL-1β production after wAIM2-targeted small interfering RNA (siRNA) interference. Six siRNAs targeting wIFI16 or wAIM2 (three each) were synthesized, and the siRNAs that reduced gene target expression by more than half were used in the subsequent silencing experiments on mRNA levels (Supplementary Fig. S1). The siRNA targeting wIFI16 (siIFI16) caused a comparable reduction in wIFI16 mRNA expression in VACV ds 70 mer-transfected and mock-infected cells (Fig. 3D). As expected, wIFN-β mRNA expression was partially inhibited in siIFI16-transfected cells with or without VACV ds 70 mer stimulation (Fig. 3D), suggesting another DNA sensor may be involved in VACV ds 70 mer-mediated IFN-β induction in woodchucks. A previous study showed that both IFI16 and the DEAD-box helicase 41 (DDX41) are responsible for VACV DNA mediated IFN-β induction in a human monocytic cell line (THP-1)^35^, while whether DDX41 or other DNA sensors are involved in woodchuck fibroblastoma cells requires further investigation. SiIFI16 inhibited wRelA and wIRF3 mRNA expression in VACV ds 70 mer-transfected cells but not in the mock-infected control cells (Fig. 3E). The siRNA targeting wAIM2 (siAIM2) decreased wAIM2 mRNA expression in both poly (dA:dT)-transfected and mock-infected cells (Fig. 3F) and blocked IL-1β mRNA production with or without poly (dA:dT) stimulation. These data indicate that VACV ds 70 mer- and poly (dA:dT)-induced wIFN-β and wIL-1β production may be mediated by wIFI16 and wAIM2, respectively.

### IFN-β and IL-1β induction by the respective ligands of wIFI16 and wAIM2 *in vivo*

**Figure 4:**
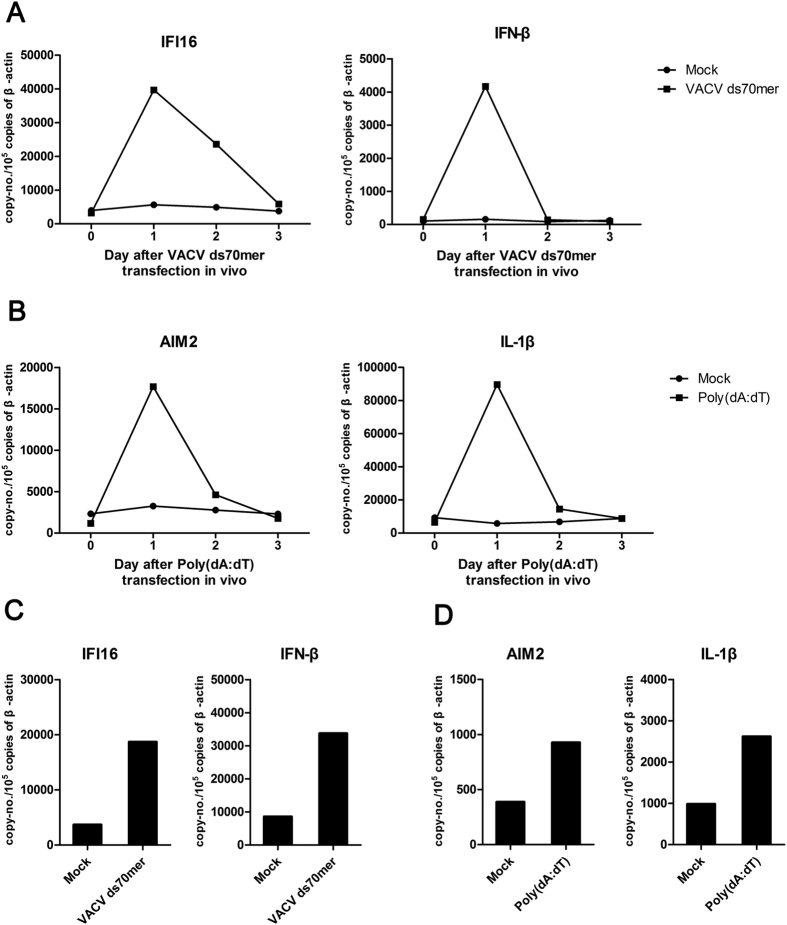
IFN-β and IL-1β induction by the respective ligands of wIFI16 and wAIM2 *in vivo*. Naïve woodchucks (n = 2) were transfected *in vivo* with VACV ds 70 mer (25 μg/kg) and poly (dA:dT) (50 μg/kg), respectively. The transcripts of wIFI16, wAIM2, wIFN-β and wIL-1β were measured by RT-qPCR of the PBMCs (**A,B**) and livers (**C,D**). The mean changes are shown.

To evaluate the effect of the respective ligands of IFI16 and wAIM2 *in vivo*, we delivered VACV ds 70 mer and poly (dA:dT) into woodchucks with the Entranster™-*in vivo* transfection reagent and measured the mRNA levels of wIFI16, wAIM2 and their respective effectors, wIFN-β and wIL-1β in peripheral blood mononuclear cells (PBMCs) and the liver. After VACV ds 70 mer *in vivo* transfection, wIFI16 and wIFN-β transcript levels increased to a maximum on day 1 in PBMCs and were upregulated on day 3 in the liver (Fig. 4A,C). After poly (dA:dT) *in vivo* transfection, wAIM2 and wIL-1β transcripts reached a peak on day 1 in PBMCs and moderately increased on day 3 in the liver (Fig. 4B,D), indicating that VACV ds 70 mer and poly (dA:dT) could induce the transcription of IFI16, wAIM2 and their respective effectors, wIFN-β and wIL-1β, *in vivo*.

### Basal wIFI16 and wAIM2 expression analysis

**Figure 5:**
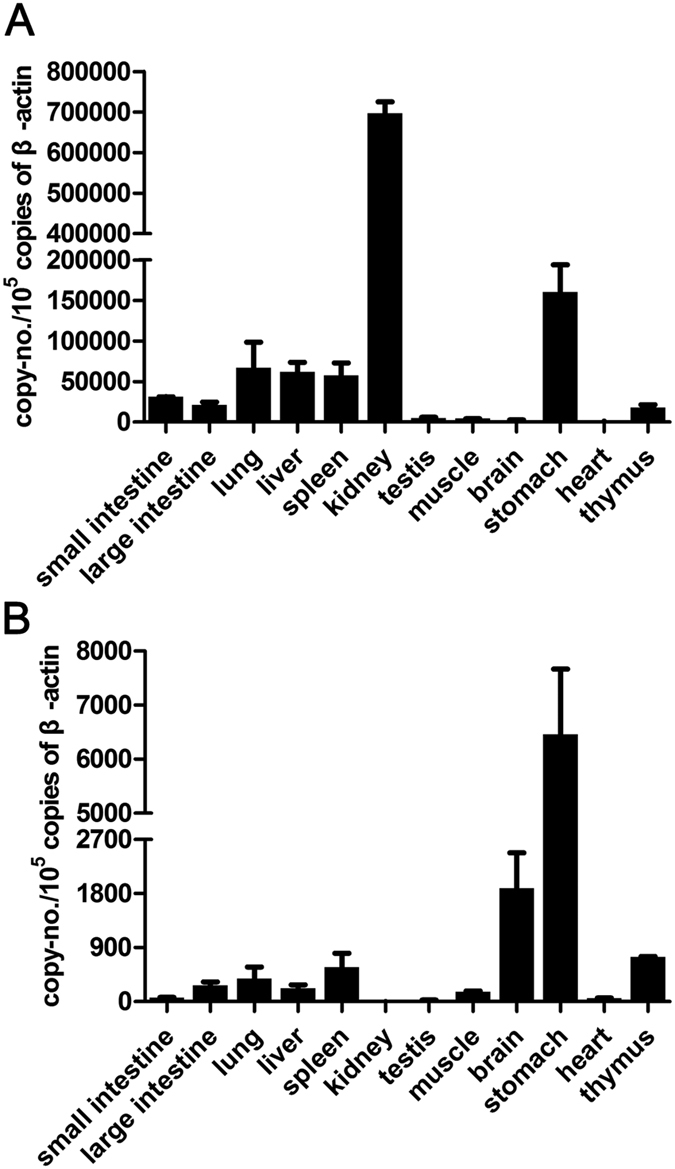
The expression of wIFI16 and wAIM2 transcripts in various organs from naïve (healthy) woodchucks. The wIFI16 (**A**) and wAIM2 (**B**) transcript expression was measured using RT-qPCR and was normalized to β-actin expression.

The samples from two naïve woodchucks were collected to investigate the transcriptional levels of wIFI16 and wAIM2 in different organs (Fig. 5). The expression of wIFI16 transcripts was highest in the kidney, followed by the stomach, liver, lung, spleen and intestine. Low-level wIFI16 expression was found in the brain, heart, muscle and testis. The expression of wAIM2 was abundant in the stomach, followed by the brain, thymus and spleen, whereas low-level wAIM2 expression was observed in the lung, liver, testis and heart. wAIM2 transcripts were not detected in the kidney.

### The expression of wIFI16 and wAIM2 in liver and spleen tissues from WHV-infected woodchucks

**Figure 6:**
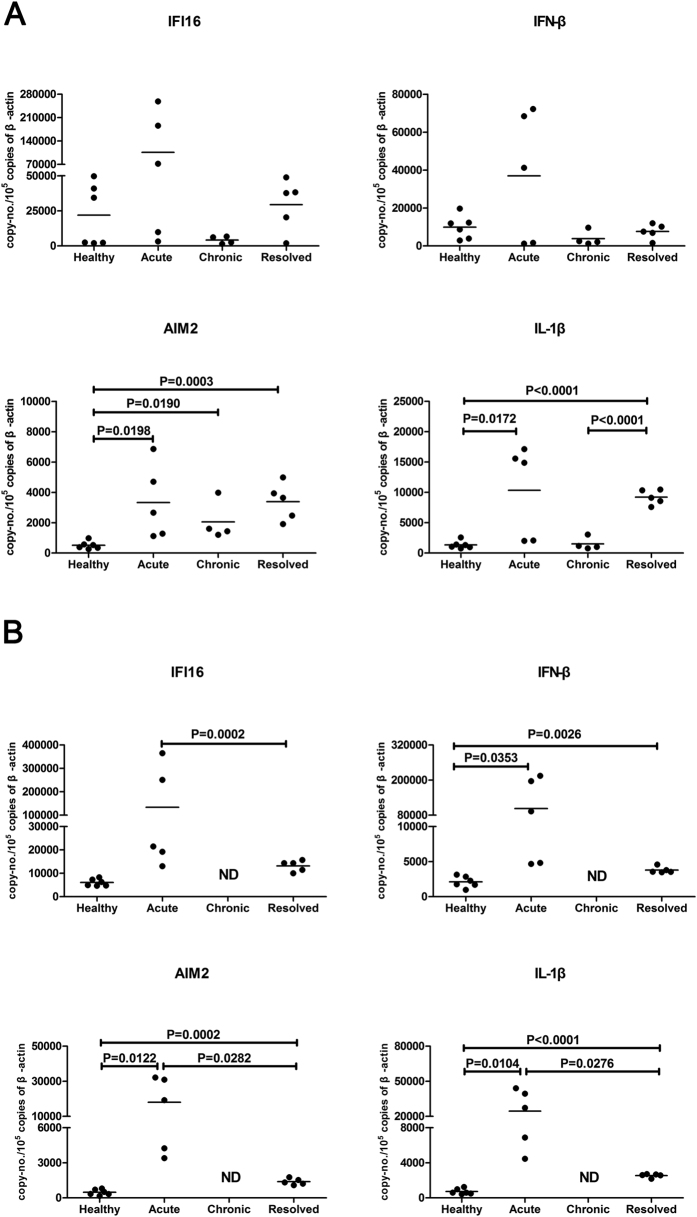
The expression of wIFI16, wAIM2 and their downstream molecules, wIFN-β and wIL-1β, in the liver and spleen of WHV-infected woodchucks. The expression of wIFI16, wAIM2, wIFN-β and wIL-1β transcripts in the liver (**A**) and spleen (**B**) of WHV-infected woodchucks were measured using RT-qPCR and were normalized to β-actin expression. The basic information of the woodchucks used in this experiment is listed in Supplementary Table S1. The data represent the averages of three independent experiments. (ND: not detected).

Twenty woodchucks were used in this study, consisting of 6 naïve (healthy) woodchucks, 5 woodchucks acutelyinfected with WHV, 5 resolved animals, and 4 woodchucks with chronic WHV infection. The descriptive statistics are listed in Supplementary Table S1. The expression levels of wIFI16 and its downstream molecule, wIFN-β, were increased in the livers and spleens from the WHV acutely infected animals, and were slightly decreased in the liver from woodchucks chronically infected with WHV, when compared with those from naïve (healthy) and/or resolved woodchucks (Fig. 6). The expression levels of wAIM2 and its downstream molecule, wIL-1β, were increased in the livers of woodchucks with acute and resolved WHV infection, and were increased in the spleens of woodchucks with acute WHV infection compared with the spleens of naïve (healthy) woodchucks (Fig. 6).

## Discussion

Experimentally WHV-infected woodchucks provide a valuable animal model for studying host-HBV interactions, but the lack of sequence information hampers functional genomics analyses in this model. To address this major limitation of the model, Fletcher *et al*. conducted a transcriptome analysis in woodchucks, providing us with a large number of woodchuck gene sequences and a valuable custom woodchuck microarray^36^,^37^. However, the intracellular innate DNA sensors IFI16 and AIM2 still require further investigation in woodchucks. In the present study, we cloned and analyzed the full-length CDSs of wIFI16 and wAIM2, examined their roles in IFN-β and IL-1β induction *in vitro*, evaluated the effect of their respective ligands *in vivo*, and measured their expression in WHV-infected woodchucks.

The deduced wIFI16 and wAIM2 proteins had 857 and 349 aa residues, respectively, and their calculated molecular weights were similar to the previously reported sizes of human and mice IFI16 and AIM2^15^,^21^,^25^. The sequence identity of wIFI16 with its orthologues from other mammalian species was as low as 30% at the aa level, while wAIM2 was relatively conserved, with a sequence identity higher than 50% at both the nt and aa levels. This result is consistent with a previous study that indicated AIM2, but not the IFI16 sequence, was highly conserved in human and mice^9^. Furthermore, we also found that both wIFI16 and wAIM2 contain the important functional structures of the ALR family, the DNA-binding HIN domain and the adaptor-binding Pyrin domain, which are conserved in human and mice^38^,^39^. In addition, wIFI16 has conserved cysteine residues (Cys801, Cys811) and a conserved MFHATV motif compared with its orthologues in other species^33^,^34^, and the M(I)FHATV and Rb-binding motifs were conserved and located at the corresponding sites in wAIM2^38^.

The basal expression of wIFI16 in the woodchuck fibroblastoma cell line was comparatively high, similar to results in human epithelial cells^40^. The VACV ds 70 mer and poly (dA:dT) are known IFI16 and AIM2 ligands, respectively, and were found to induce wIFN-β and wIL-1β expression, respectively, in human and mouse cells^17^,^20^,^21^,^22^,^23^. Consistently, wIFI16 and wIFN-β transcripts were upregulated after VACV ds 70 mer transfection in woodchuck cells, while wAIM2 and wIL-1β transcripts were also upregulated after poly (dA:dT) transfection. SiRNA silencing of wIFI16 partially inhibits VACV ds 70 mer-stimulated wIFN-β transcription, and wAIM2 silencing inhibits poly (dA:dT)-induced wIL-1β transcription, indicating that the induction of wIFN-β by VACV ds 70 is partially dependent on wIFI16, while wAIM2 is responsible for poly (dA:dT)-mediated-wIL-1β induction. In addition, we found that wIFI16 and wIFN-β transcripts were upregulated after VACV ds 70 mer *in vivo* transfection, and wAIM2 and wIL-1β transcripts were also upregulated after poly (dA:dT) *in vivo* transfection, providing the basis for using wIFI16 and wAIM2 ligands, poly (dA:dT) and VACV ds 70 mer, in the woodchuck model.

The expression of wIFI16 transcripts was abundant in the kidney, stomach, liver, lung and spleen, in which a variety of lymphocytes was colonized as a result of persistent contact with exogenous DNA. Similarly, IFI16 was primarily detected in the lymphoid tissues and in human epithelial cells^40^. A previous study showed that AIM2 plays a critical role in autoimmune diseases of the nervous system and repair^41^,^42^; as expected, wAIM2 transcripts were also highly expressed in the woodchuck brain and stomach.

IFI16 and AIM2 play important roles in controlling viral replication^18^,^19^. Therefore, we investigated the expression of wIFI16, wAIM2 and their downstream molecules, wIFN-β and wIL-1β in the liver (the primary target organ of HBV) and/or the spleen (an important immune organ) of woodchucks infected with WHV. We found that both wIFI16 and its downstream molecule, wIFN-β were upregulated in woodchucks acutely infected with WHV and were slightly downregulated in those with chronic infection. The expressions of wAIM2 and its downstream molecule, wIL-1β were increased in woodchucks with acute and resolved WHV infection but remained almost unchanged in those with chronic infection. This observation is not completely consistent with a previous study in woodchucks. Using microarray analysis, Fletcher *et al*. found that IFI16 and AIM-2 were increased in the livers of woodchucks with acute and chronic infection, but not in resolved WHV infection^37^. However, our observation is consistent with the findings in humans, in which AIM2 transcript levels were found to be increased in PBMCs from acutely HBV-infected patients but remained nearly unchanged in chronically HBV-infected patients^43^. Taken together, the differential expression pattern of IFI16 and AIM2 transcripts following WHV/HBV infection implied that these two DNA sensors might be involved in WHV/HBV infection. Further investigation, e.g., using a knockdown strategy, is needed to verify whether IFI16 and AIM2 are essential for controlling WHV/HBV infection.

In conclusion, we identified the DNA sensors IFI16 and AIM2 and their ligands in a woodchuck model, which is a valuable model for studying HBV infection. Our data also imply the possible role of IFI16 and AIM2 in WHV/HBV infection, which is useful for further investigation. These results provide the basis for the further study of IFI16- and AIM2-mediated innate immunity in HBV infection using the informative woodchuck model.

## Methods

### Ethics statement

American woodchucks were purchased from the Institute of Laboratory Animal Sciences of the Chinese Academy of Medical Sciences and were maintained at the Experimental Animal Center of Huazhong University of Science and Technology. Chinese woodchucks were captured from Qinghai province, China, and were maintained at the Experimental Animal Center of Qinghai. All efforts were made to alleviate stress and any incidental deaths. The woodchucks were anesthetized via intramuscular ketamine injection. PBMCs were isolated from the blood. Liver and spleen specimens were obtained from the Chinese woodchuck sample pool in our lab. The experiments were conducted in accordance with the Guide for the Care and Use of Laboratory Animals (National Academy Press, revised 1996) and were reviewed and approved by the local Animal Care and Use Committees (Animal Ethics Committee of Tongji Medical College, Huazhong University of Science and Technology, China).

### Cloning and the sequence analysis of wIFI16 and wAIM2

Woodchuck PBMCs were isolated and stimulated with 5 μg/ml concanavalin A (ConA) (Sigma, St. Louis, MO, USA) for 24 hours. Total RNA was extracted using TRIzol (Takara, Dalian, China) according to the manufacturer’s instructions and was used as the template for subsequent complementary DNA (cDNA) synthesis. The synthesized cDNA was used as a template for the PCR amplification of the CDSs of wIFI16 and wAIM2. The primers used to generate the wIFI16 and wAIM2 CDSs are listed in Supplementary Table S2. The resulting PCR products were cloned into the pMDT-18 vector according to the manufacturer’s instructions (Takara), and the plasmids containing wIFI16 or wAIM2 were sequenced via a commercial service (Invitrogen, Carlsbad, CA, USA).

Multiple sequence alignment was performed using DNAMAN (Lynnon Biosoft Corporation, San Ramon, CA, USA). The protein domain prediction was performed using the CD Search program (http://www.ncbi.nlm.nih.gov/Structure/cdd/wrpsb.cgi). Phylogenetic trees were constructed using the neighbor-joining (NJ) method with Mega 4.1^44^.

### Woodchuck IFI16 and AIM2 ligands stimulation *in vitro* and *in vivo* and siRNA interference

The WH12/6 cell line was kindly provided by P. Banasch of the German Cancer Research Center, DKFZ Heidelberg, Germany, and maintained in Ham’s F12 medium supplemented with 10% FBS (Gibco, Carlsbad, CA, USA) and 1% penicillin/streptomycin (Gibco), as described previously^45^.

The VACV ds 70 mer, the VACV ss 70 mer and poly (dA:dT) (InvivoGen, SanDiego, CA, USA) were transfected into WH12/6 cells at a concentration of 2 μg/ml for 6 hours using Lipofectamine 2000 (Invitrogen) according to the manufacturer’s instructions. After 18 hours, the cells were harvested, snap-frozen in liquid nitrogen, and then stored at −80 °C until RNA extraction.

The siRNAs (double-stranded RNA oligonucleotides) and negative control duplexes were chemically synthesized (Invitrogen). The siRNA sequences are listed in Supplementary Table S3. WH12/6 cells were transfected with 100 nM Silencer Select siRNAs for wIFI16 or wAIM2 and the negative control duplexes and were subsequently transfected with the VACV ds 70 mer, the VACV ss 70 mer or poly (dA:dT) at a concentration of 2 μg/ml using Lipofectamine 2000 (*In vitro* gen) according to the manufacturer’s instructions. After 18 hours, the cells were harvested, snap-frozen in liquid nitrogen, and then stored at −80 °C until RNA extraction.

The VACV ds 70 mer and poly (dA:dT) (*In vivo* Gen, SanDiego, CA, USA) were administered into naïve woodchucks (n = 2) at doses of 25 μg/kg and 50 μg/kg, respectively, using the Entranster™-*in vivo* transfection reagent (Engreen Biosystem Co. Beijing, China) according to the manufacturer’s instructions. PBMCs were isolated from the whole blood at day0, day1, day2 and day3 after ligands administration, while the livers were collected at day3 after ligands administration, snap-frozen in liquid nitrogen, and then stored at −80 °C until RNA extraction.

### Sample collection, RNA extraction and reverse transcription quantitative real-time PCR (RT-qPCR)

Small intestine, large intestine, lung, liver, spleen, kidney, testis, muscle, brain, stomach, heart and thymus specimens from 2 naïve (healthy) woodchucks and liver and spleen samples from 16 woodchucks were collected by necropsy, snap-frozen in liquid nitrogen, and stored at −80 °C until RNA extraction.

Total RNA was extracted from WH12/6 cells and tissue samples using TRIzol (Takara) according to the manufacturer’s instruction. The quality and quantity of the RNAs were evaluated by calculating the A260/A280 ratio and 28 S/18 S ribosomal RNA ratio. RT-qPCR was conducted using the RNA templates and the SYBR Green I Kit (Toyobo, Dalian, China) according to the manufacturer’s instructions in the CFX Connect™ Real-Time PCR Detection System (BIO-RAD, Hercules, California, USA). RT-qPCR was carried out in a 20 μL volume, containing 10 μL of 2 × Buffer (dNTP mixture, Mg^2 + ^and SYBR Green I), 0.8 μL of enzyme mix (RTase, RNase inhibitor and Ex Taq), 0.8 μL of 10 μΜ forward primers, 0.8 μL of 10 μΜ reverse primers, 2 μL of 50 ng/μL total RNA, and 5.6 μL of RNase free dH_2_O. The cycling parameters were 42 °C for 5 min, 95 °C for 10 s, followed by 40 cycles of 5 s at 95 °C and 30 s at 60 °C. Primer sets were designed using the Primer Express^TM^ 3.0 software (Applied Biosystems, Carlsbad, CA, USA). All these primer pairs produced a single amplicon according to the specific PCR product seen upon 2% agar gel electrophoresis and the single peak dissociation curves of RT-qPCR products. The primers used for the RT-qPCR of wIFI16, wAIM2, wIFN-β, wRelA, wIRF3, wIL-1β and β-actin are listed in Supplementary Table S2. The primer efficiencies were evaluated by the calibration curve generated with serial dilutions (covering 3 orders of magnitude) of cDNA (Supplementary Fig. S2). The efficiency of all primer pairs ranged from 0.91 to 1.09. The R^2^ values (correlation coefficients) were between 0.990 and 0.996. No-template controls were included to ensure the absence of reagent contamination and genomic DNA. All of the reactions were performed in triplicate, and the mean value of the quantification cycle (Cq) was used for subsequent analysis. The quantification was performed using the Pfaffl method^46^ by calculating the copy numbers from the Cq-value for each gene per sample. Beta-actin expression was used to normalize the mRNA expression levels of wIFI16, wAIM2, wIFN-β, wRelA, wIRF3 and wIL-1β^47^,^48^,^49^, and the expression levels were given as the copy number/100,000 copies of β-actin mRNA.

### Statistical analysis

SPSS18.0 (IBM Corporation, Somers, NY, USA) was used to assess the statistical significance of differences (*p*-values) between the groups using unpaired t-tests with equal variance. A *p*-value < 0.05 was considered significant.

## Additional Information

**How to cite this article**: Yan, Q. *et al*. Molecular characterization of woodchuck IFI16 and AIM2 and their expression in woodchucks infected with woodchuck hepatitis virus (WHV). *Sci. Rep*. **6**, 28776; doi: 10.1038/srep28776 (2016).

## Supplementary Material

Supplementary Information

## Figures and Tables

**Table 1:** The identities of wIFI16 with the orthologues of the other species.

Species	Common name	Accession no.	Identity(%)	
			nt	aa
*Homo sapiens*	Human	BC017059	55.23	36.31
*Pan troglodytes*	Chimpanzee	XM_001170378	54.67	36.31
*Mus musculus*	House mouse	NM_008329	53.13	32.33
*Equus caballus*	Horse	XM_005609978	76.26	64.22
*Leptonychotes weddellii*	Weddell seal	XM_006748515	66.95	49.15
*Bos taurus*	Cattle	XM_002685877	61.67	44.04
*Felis catus*	Cat	XM_006943050	62.17	39.73
*Canis lupus*	Dog	XM_005640909	62.61	45.40
*Panthera tigris altaica*	Tiger	XM_007084298	60.05	36.39
*Elephantulus edwardii*	Elephant	XM_006895523	52.53	29.27
*Odobenus rosmarus divergens*	Walrus	XM_004407844	75.83	65.33

**Table 2:** The identities of wAIM2 with the orthologues of the other species.

Species	Common name	Accession no.	Identity(%)	
			nt	aa
*Homo sapiens*	Human	NM_004833	77.49	68.91
*Pan troglodytes*	Chimpanzee	XM_009435232	78.14	70.09
*Mus musculus*	House mouse	NM_001013779	70.62	56.81
*Equus caballus*	Horse	XM_005609975	78.11	68.99
*Leptonychotes weddellii*	Weddell seal	XM_006752400	63.58	52.80
*Tupaia chinensis*	Chinese tree shrew	XM_006168874	75.19	66.57
*Rattus norvegicus*	Norway rat	XM_222949	68.87	54.76
