# Supplementary material for: Molecular characterization of woodchuck IFI16 and AIM2 and their expression in woodchucks infected with woodchuck hepatitis virus (WHV)
Source: Sci Rep. 2016 Jun 29;6:28776. doi: 10.1038/srep28776 (PMC4926060; doi:10.1038/srep28776)
Supplement: Supplementary Information [file srep28776-s1.pdf]

Molecular characterization of woodchuck IFI16 and AIM2 and their expression in woodchucks infected with woodchuck hepatitis virus (WHV)

Qi Yan[1], Mengmeng Li[1], Qin Liu[1], Fanghui Li[1], Bin Zhu[1], Junzhong Wang[1], Yinping Lu[1], Jia Liu[1,2], Jun Wu[1], Xin Zheng[1], Mengji Lu[2], Baoju Wang[1]*, Dongliang Yang[1]*

1 Department of Infectious Diseases, Union Hospital, Tongji Medical College, Huazhong University of Science and Technology, Wuhan, China

2 Institute of Virology, University of Duisburg-Essen, Essen, Germany

Qi Yan

E-mail: qi10yan@163.com

Mengmeng Li

E-mail: lemengle891227@163.com

Qin Liu

E-mail: fightingliuqin@163.com

Fanghui Li

E-mail: 15927580042@163.com

Bin Zhu

E-mail: fuyuanzhubin@163.com

Junzhong Wang

E-mail: wang_junzhong@163.com

Yinping Lu

E-mail: yinpinglu@163.com

Jia Liu

E-mail: bug_lj@163.com
Jun Wu
E-mail: 18627006732@163.com
Xin Zheng
E-mail: xin11@hotmail.com
Mengji Lu
mengji.lu@uni-due.de

*Co-corresponding author
Baoju Wang, Ph. D.
Associate Professor
Department of Infectious Diseases
Union Hospital of Tongji Medical College
Huazhong University of Science and Technology
1277# Jiefang Avenue
430022, Wuhan, China
E-mail: bjwang73@163.com;

Dongliang Yang, Ph. D.
Professor
Department of Infectious Diseases
Union Hospital of Tongji Medical College
Huazhong University of Science and Technology
1277# Jiefang Avenue
430022, Wuhan, China

E-mail: dlyang55@hotmail.com

Supplementary materials

Supplementary Table S1. The basic information of the woodchucks enrolled in this study.

Supplementary Table S2. Primers used for cloning and RT-qPCR

Supplementary Table S3. Small interference RNA sequences used in this study.

Supplementary Fig. S1. The effects of siRNAs on the expression of wIFI16 and wAIM2 in WH12/6 cells. Different siRNAs targeting wIFI16 (A) or wAIM2 (B) were transfected into WH12/6 cells. The cells were collected after 24 hours, and the mRNA expression levels of wIFI16 and wAIM2 were measured using RT-qPCR and normalized with β-actin expression.

Supplementary Fig. S2. Determination of PCR efficiencies of wIFI16(A), IFN-β (B), RelA(C), IRF3(D), wAIM2(E), IL-1β(F) and β-actin(G). The initial cDNA (reverse transcripts from total RNA) concentration ($\log_{10}$) was plotted on the *x* axis and Cq value was plotted on the *y* axisto calculate the slope (mean±SD; n=3),*y* interceptand correlation coefficient (R^2). PCR efficiency (E)=$10^{-1/slope}$-1.

Table S1. The basic information of the woodchucks enrolled in this study.

Group	Number	Age (year)	Sex	Viral load (copies /ml)	Duration of infection (week)
Healthy	2889	2-3	Male		
	2893	3	Male		
	2873	2-3	Male		
	2895	2-3	Male		
	2732	2-3	Female		
	2800	2-3	Female		
Acute	1003	2-3	Male	3.44×10^{8}	8
	1023	2-3	Female	6.49×10^{10}	12
	0407	2-3	Male	2.56×10^{6}	10
	0409	2-3	Female	3.46×10^{6}	14
	TR0410	2-3	Female	1.3×10^{6}	11
Chronic	4850	2-3	Female	1.66×10^{9}	>52
	4854	2-3	Female	3.26×10^{8}	>52
	4856	2-3	Male	1.28×10^{9}	>52
	6186	2-3	Male	1.21×10^{9}	>52
Resolved	1007	2-3	Male		12
	0402	2-3	Female		14
	0403	2-3	Male		9
	0408	2-3	Female		12
	TGL0410	2-3	Female		13

Table S2. Primers used for cloning and RT-qPCR

Primer	Accession number	Polarity	Nucleotide sequence	Amplicon Length (bp)	nt Position
wIFI16-s	XM_005339423	Sense	5'-GAAATGGTGAACGAATACAA-3'	2580	-3-17
wIFI16-as		Antisense	5'-CTTTTAGGAGGATTCTTGTG-3'		2558-2577
wAIM2-s	XM_005339421	Sense	5'-ATGGAGAGCAAATACAGAGAAATGCT-3'	1050	1-26
wAIM2-as		Antisense	5'-TTACTTTTTGGCCTTAACAACCGTA-3'		1026-1050
qwIFI16-s	KP334127	Sense	5'-GAAGGACAAATTCATCCCAAAG-3'	80	2082-2103
qwIFI16-as		Antisense	5'-CACTGTATAGCTCCAGGAACCC-3'		2140-2161
qwAIM2-s	KP272148	Sense	5'-CTAAGCAGAAGCAGGTGA-3'	118	416-433
qwAIM2-as		Antisense	5'-TCTTGGGTCTCAAACTCG-3'		516-533
qIFN-β-s	DQ402072	Sense	5'-TCTCCACCACAGCTCTTTCC-3'	103	39-58
qIFN-β-as		Antisense	5'-GGCCTTTCATTCAACTGCTCT-3'		121-141
qRelA-s		Sense	5'-GGCTTCTATGAGGCTGAG-3'		
qRelA-as		Antisense	5'-GTTGTTGTTGGTCTGGATG-3'		
qIRF3-s	EU586557	Sense	5'-GGTGAAGAGGCTTGTGATGG-3’	125	1071-1090
qIRF3-as		Antisense	5'-GGTGGCTGTTGGAAATGTGT-3'		1176-1195
qIL-1β-s	EU564729	Sense	5'-GACCGAATCTGAGGCAACAA-3'	134	162-181
qIL-1β-as		Antisense	5'-CTTGTCCCCCTTCATCACAC-3'		276-295
qβ-actin-s	AY170121	Sense	5'-TGGAATCCTGTGGCATCCATGAAAC -3'	346	1-24
qβ-actin-as		Antisense	5'-TAAAACGCAGCTCAGTAACAGTCCG -3'		322-346

Notes: The primers of wIFI16 and wAIM2 were used for cloning. The primers of qwIFI16, qwAIM2, qIFN-β, qRelA, qIRF3, qIL-1β and qβ-actin were used for RT-qPCR.

Table S3. Small interference RNA sequences used in this study

siRNA	Polarity	Nucleotide sequence
siAIM2#1-s	Sense	5'-GGUGCUGAAAGCAAUGAAGTT-3'
siAIM2#1-as	Antisense	5'-CUUCAUUGCUUUCAGCACCTT-3'
siAIM2#2-s	Sense	5'-GCAAUGAAGCCCUUCGAGUTT-3'
siAIM2#2-as	Antisense	5'-ACUCGAAGGGCUUCAUUGCTT-3'
siAIM2#3-s	Sense	5'-GAAGAAAGCGAGAGAUGUUTT-3'
siAIM2#3-as	Antisense	5'-AACAUCUCUCGCUUUCUUCTT-3'
siIFI16#1-s	Sense	5'-CCACUGAGAGCGAAUUCUUTT-3'
siIFI16#1-as	Antisense	5'- AAGAAUUCGCUCUCAGUGGTT-3'
siIFI16#2-s	Sense	5'- CCGAUUACAUUGGCCGCAATT-3'
siIFI16#2-as	Antisense	5' -UUGCGGCCAAUGUAAUCGGTT-3'
siIFI16#3-s	Sense	5' -GAGUAUCUUUGCUCAAGAATT-3'
siIFI16#3-as	Antisense	5' -UUCUUGAGCAAAGAUACUCTT-3'

FIG S1

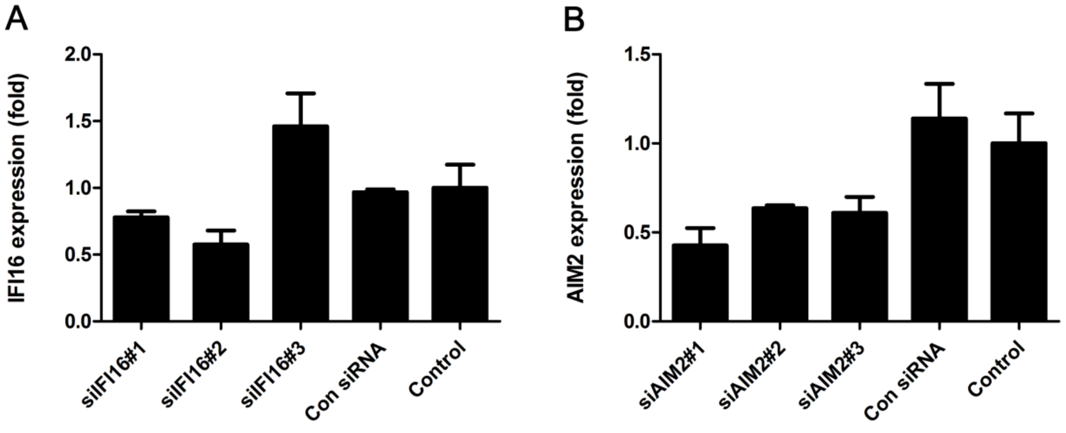
A
IFI16 expression (fold)
2.0
1.5
1.0
0.5
0.0
siIFI16#1
siIFI16#2
siIFI16#3
Con siRNA
Control
B
AIM2 expression (fold)
1.5
1.0
0.5
0.0
siAIM2#1
siAIM2#2
siAIM2#3
Con siRNA
Control

FIG S2

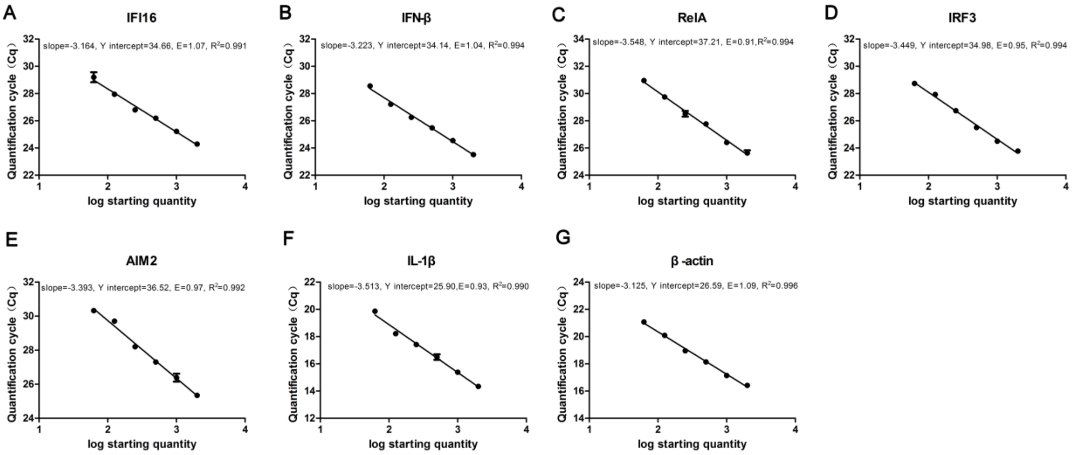
A
IFI16
slope=-3.164, Y intercept=34.66, E=1.07, R²=0.991
B
IFN-β
slope=-3.223, Y intercept=34.14, E=1.04, R²=0.994
C
RelA
slope=-3.548, Y intercept=37.21, E=0.91,R²=0.994
D
IRF3
slope=-3.449, Y intercept=34.98, E=0.95, R²=0.994
E
AIM2
slope=-3.393, Y intercept=36.52, E=0.97, R²=0.992
F
IL-1β
slope=-3.513, Y intercept=25.90,E=0.93, R²=0.990
G
β -actin
slope=-3.125, Y intercept=26.59, E=1.09, R²=0.996
Quantification cycle（Cq）
log starting quantity
